# Supplementary material for: From library to landscape: integrative annotation workflows for compound libraries in drug repurposing
Source: Database (Oxford). 2025 Dec 9;2025:baaf081. doi: 10.1093/database/baaf081 (PMC12687465; doi:10.1093/database/baaf081)
Supplement: baaf081_Supplemental_Files [file baaf081_supplemental_files.zip › R4A-annotation-manuscript-DATABASE-SupplementaryData-noFigs_JR.docx]

Supplementary Data

**From Library to Landscape: Integrative Annotation Workflows for Compound Libraries in Drug Repurposing**

Jeanette Reinshagen^1^, Brinton Seashore-Ludlow^2,3^, Yojana Gadiya^1,4^, Anna-Lena Gustavsson^3^, Ziaurrehman Tanoli^5^, Tero Aittokallio^5,6,7^, Johanna Huchting^1^, Annika Jenmalm-Jensen^3^, Philip Gribbon^1^, Andrea Zaliani^1^, Flavio Ballante^*,3^

**Affiliations**:

1. Fraunhofer Institute for Translational Medicine and Pharmacology (ITMP), Hamburg 22525, Germany
2. Department of Oncology-Pathology, Karolinska Institutet, Science for Life Laboratory, Solna, Sweden
3. Chemical Biology Consortium Sweden (CBCS), Science for Life Laboratory, Department of Medical Biochemistry and Biophysics, Karolinska Institutet, Stockholm, Sweden
4. Bonn-Aachen International Center for Information Technology (B-IT), University of Bonn, 53113 Bonn, Germany
5. Institute for Molecular Medicine Finland (FIMM), HiLIFE, University of Helsinki, Helsinki, Finland
6. Institute for Cancer Research, Department of Cancer Genetics, Oslo University Hospital, Oslo, Norway
7. Oslo Centre for Biostatistics and Epidemiology (OCBE), Faculty of Medicine, University of Oslo, Oslo, Norway

***Corresponding author:**

Flavio Ballante ([flavio.ballante@ki.se](mailto:flavio.ballante@ki.se))

Number of pages: 4

Number of tables: 3

Number of figures: 5

**Table of content**

[**Tables** 3](#_Toc205972130)

[**Table S1** 3](#_Toc205972131)

[**Table S2** 4](#_Toc205972132)

[**Table S3** 4](#_Toc205972133)

[**Figures** 4](#_Toc205972134)

[**Figure S1** 4](#_Toc205972135)

[**Figure S2** 4](#_Toc205972136)

[**Figure S3** 4](#_Toc205972137)

[**Figure S4** 4](#_Toc205972138)

[**Figure S5** 4](#_Toc205972139)

# **Tables**

**Table S1.** Compound-centric information retrieved by the KNIME and Python annotation pipelines**.**

| **Description** | **KNIME annotation** | **Python annotation** | **Overlap** | **Jaccard index^a^** |
| --- | --- | --- | --- | --- |
| Total library size | 5254 | 5254 | 5254 | 1 |
| Unique compounds after structure harmonisation | 5230 | 5230 | 5230 | 1 |
| CID | 5091 | 5217 | 4592 | 0.8 |
| sChEMBL ID | 4585 | 4787 | 4296 | 0.85 |
| ChEMBL ID | 4688 | 4954 | 4457 | 0.86 |
| Launched | 1155 | 1304 | 1113 | 0.83 |
| Phase 3 | 292 | 302 | 278 | 0.88 |
| Phase 2 | 790 | 812 | 772 | 0.93 |
| Phase 1 | 131 | 133 | 126 | 0.91 |
| Early Phase 1 | 3 | 3 | 3 | 1 |
| Unknown phase | 148 | 150 | 141 | 0.9 |
| Preclinical/discovery | 2169 | 2523 | 2098 | 0.81 |
| Withdrawn | 81 | 90 | 79 | 0.86 |
| clinical trials (total) | 1216 | 1250 | 1179 | 0.92 |
| Compounds with known MoA | 1209 | 1280 | 1156 | 0.87 |
| Compounds with known indication class | 983 | 1114 | 948 | 0.83 |
| Compounds with EFO term | 1851 | 2013 | 1786 | 0.86 |
| Compounds with Mesh heading | 1851 | 2013 | 1786 | 0.86 |
| ^a^ Jaccard Index = $\frac{Ni}{\left( Na+Nb-Ni \right)}$ | | | | |

| Table S2. Annotation-centric information retrieved by the KNIME and Python annotation pipelines. Constrains: pChembl ≥ 6, Confidence score ≥ 8 | | | | |
| --- | --- | --- | --- | --- |
| Description | **KNIME Annotation** | **Python Annotation** | **Overlap** | **Jaccard Index ^a^** |
| Number of MoA | 630 | 647 | 619 | 0.94 |
| Number of indication class | 348 | 389 | 342 | 0.87 |
| Number of EFO term | 1760 | 1870 | 1739 | 0.92 |
| Number of Mesh heading | 1507 | 1599 | 1489 | 0.92 |
| Endpoints (pChEMBL) | 59751 | 60597 | 57923 | 0.93 |
| Targets | 2202 | 2295 | 2172 | 0.93 |
| Assays | 33913 | 36492 | 33042 | 0.88 |
| ^a^ Jaccard Index =$\frac{\boldsymbol{Ni}}{\left( \boldsymbol{Na+Nb-Ni} \right)}$ | | | | |

**Table S3.** Target hierarchy counts from the KNIME and Python workflows. Table is available as supplementary file TableS3.pdf.

# **Figures**

| **Figure S1.** Directory structure of the Python annotation pipeline. Comments on the right provide a brief explanation of each file. |
| --- |

| **Figure S2.** Chemical space coverage of R4A set (orange) compared to ChEMBL clinical compounds (blue). Differences in sizes of squares are for better visualization only and do not reflect sector size or compound count. |
| --- |

| **Figure S3.** Chemical space coverage of R4A set (orange) compared to Drug repurposing hub (yellow). Differences in sizes of squares are for better visualization only and do not reflect sector size or compound count. |
| --- |

| **Figure S4.** Top-ranked EFO terms based on n of unique compounds. Data from binding and functional assays with a pChEMBL value ≥ 6 and a confidence score ≥ 8 were considered. |
| --- |

| **Figure S5.** Overlap of protein targets between the R4A set and ChEMBL, grouped by target class. a) Overlap of UniProt IDs for targets associated with the molecules (ChEMBL IDs); b) Overlap of UniProt IDs in Homo sapiens. Count is based on the number of unique UniProt IDs, considering binding and functional assays with a pChEMBL value ≥ 6 and a confidence score ≥ 8. The data shown was obtained from the Python pipeline. |
| --- |
