# Supplementary material for: From library to landscape: integrative annotation workflows for compound libraries in drug repurposing
Source: Database (Oxford). 2025 Dec 9;2025:baaf081. doi: 10.1093/database/baaf081 (PMC12687465; doi:10.1093/database/baaf081)
Supplement: baaf081_Supplemental_Files [file baaf081_supplemental_files.zip › TableS3.pdf]

Index	Level1	Level2	Level3	Level4	Level5	Level6	Python_ Workflow	Knime_ Workflow
1	Adhesion						7	6
2	Auxiliary transport protein						2	1
3	Auxiliary transport protein	Calcium channel auxiliary subunit alpha2delta family					6	3
4	Auxiliary transport protein	Fatty acid binding protein family					11	9
5	Enzyme						167	156
6	Enzyme	Aminoacyltransferase	Protein-glutamine glutamyl-transferase				1	1
7	Enzyme	Cytochrome P450					3	3
8	Enzyme	Cytochrome P450	Cytochrome P450 family 1	Cytochrome P450 family 1A	Cytochrome P450 1A1		2	2
9	Enzyme	Cytochrome P450	Cytochrome P450 family 1	Cytochrome P450 family 1A	Cytochrome P450 1A2		114	115
10	Enzyme	Cytochrome P450	Cytochrome P450 family 1	Cytochrome P450 family 1B	Cytochrome P450 1B1		12	12
11	Enzyme	Cytochrome P450	Cytochrome P450 family 11	Cytochrome P450 family 11B	Cytochrome P450 11B1		1	2
12	Enzyme	Cytochrome P450	Cytochrome P450 family 11	Cytochrome P450 family 11B	Cytochrome P450 11B2		1	2
13	Enzyme	Cytochrome P450	Cytochrome P450 family 17	Cytochrome P450 family 17A	Cytochrome P450 17A1		12	12
14	Enzyme	Cytochrome P450	Cytochrome P450 family 19	Cytochrome P450 family 19A	Cytochrome P450 19A1		18	19
15	Enzyme	Cytochrome P450	Cytochrome P450 family 2	Cytochrome P450 family 2A	Cytochrome P450 2A6		1	
16	Enzyme	Cytochrome P450	Cytochrome P450 family 2	Cytochrome P450 family 2C	Cytochrome P450 2C19		63	63
17	Enzyme	Cytochrome P450	Cytochrome P450 family 2	Cytochrome P450 family 2C	Cytochrome P450 2C9		43	39
18	Enzyme	Cytochrome P450	Cytochrome P450 family 2	Cytochrome P450 family 2D	Cytochrome P450 2D6		96	94
19	Enzyme	Cytochrome P450	Cytochrome P450 family 24	Cytochrome P450 family 24A	Cytochrome P450 24A1		1	1
20	Enzyme	Cytochrome P450	Cytochrome P450 family 26				2	2
21	Enzyme	Cytochrome P450	Cytochrome P450 family 26	Cytochrome P450 family 26A	Cytochrome P450 26A1		3	3
22	Enzyme	Cytochrome P450	Cytochrome P450 family 3	Cytochrome P450 family 3A	Cytochrome P450 3A4		47	47
23	Enzyme	Cytochrome P450	Cytochrome P450 family 3	Cytochrome P450 family 3A	Cytochrome P450 3A5		1	1
24	Enzyme	Cytochrome P450	Cytochrome P450 family 5	Cytochrome P450 family 5A	Cytochrome P450 5A1		15	15
25	Enzyme	Cytochrome P450	Cytochrome P450 family 51	Cytochrome P450 family 51A	Cytochrome P450 51A1		14	14
26	Enzyme	Hydrolase					306	272
27	Enzyme	Isomerase					66	51
28	Enzyme	Kinase					28	23
29	Enzyme	Kinase	Protein Kinase				33	31
30	Enzyme	Kinase	Protein Kinase	AGC protein kinase group	AGC protein kinase AKT family		45	41
31	Enzyme	Kinase	Protein Kinase	AGC protein kinase group	AGC protein kinase DMPK family	AGC protein kinase CRIK subfamily	18	17
32	Enzyme	Kinase	Protein Kinase	AGC protein kinase group	AGC protein kinase DMPK family	AGC protein kinase GEK subfamily	24	23
33	Enzyme	Kinase	Protein Kinase	AGC protein kinase group	AGC protein kinase DMPK family	AGC protein kinase ROCK subfamily	76	72
34	Enzyme	Kinase	Protein Kinase	AGC protein kinase group	AGC protein kinase GRK family	AGC protein kinase BARK subfamily	2	2
35	Enzyme	Kinase	Protein Kinase	AGC protein kinase group	AGC protein kinase GRK family	AGC protein kinase GRK subfamily	34	32
36	Enzyme	Kinase	Protein Kinase	AGC protein kinase group	AGC protein kinase MAST family		5	5
37	Enzyme	Kinase	Protein Kinase	AGC protein kinase group	AGC protein kinase NDR family		24	20
38	Enzyme	Kinase	Protein Kinase	AGC protein kinase group	AGC protein kinase PDK1 subfamily		12	13
39	Enzyme	Kinase	Protein Kinase	AGC protein kinase group	AGC protein kinase PKA family		47	47
40	Enzyme	Kinase	Protein Kinase	AGC protein kinase group	AGC protein kinase PKC family	AGC protein kinase PKC alpha subfamily	32	23
41	Enzyme	Kinase	Protein Kinase	AGC protein kinase group	AGC protein kinase PKC family	AGC protein kinase PKC delta subfamily	39	38
42	Enzyme	Kinase	Protein Kinase	AGC protein kinase group	AGC protein kinase PKC family	AGC protein kinase PKC eta subfamily	19	18
43	Enzyme	Kinase	Protein Kinase	AGC protein kinase group	AGC protein kinase PKC family	AGC protein kinase PKC iota subfamily	6	6
44	Enzyme	Kinase	Protein Kinase	AGC protein kinase group	AGC protein kinase PKG family		22	22
45	Enzyme	Kinase	Protein Kinase	AGC protein kinase group	AGC protein kinase PKN family		44	41
46	Enzyme	Kinase	Protein Kinase	AGC protein kinase group	AGC protein kinase RSK family	AGC protein kinase MSK subfamily	37	31
47	Enzyme	Kinase	Protein Kinase	AGC protein kinase group	AGC protein kinase RSK family	AGC protein kinase RSK subfamily	94	86
48	Enzyme	Kinase	Protein Kinase	AGC protein kinase group	AGC protein kinase RSK family	AGC protein kinase p70 subfamily	26	26
49	Enzyme	Kinase	Protein Kinase	AGC protein kinase group	AGC protein kinase SGK family		12	11
50	Enzyme	Kinase	Protein Kinase	AGC protein kinase group	AGC protein kinase YANK family		6	6
51	Enzyme	Kinase	Protein Kinase	Atypical protein kinase group			1	1

Index	Level1	Level2	Level3	Level4	Level5	Level6	Python_ Workflow	Knime_ Workflow
52	Enzyme	Kinase	Protein Kinase	Atypical protein kinase group	Atypical protein kinase ABC1 family	Atypical protein kinase ABC1-A subfamily	3	3
53	Enzyme	Kinase	Protein Kinase	Atypical protein kinase group	Atypical protein kinase PDHK subfamily		3	4
54	Enzyme	Kinase	Protein Kinase	Atypical protein kinase group	Atypical protein kinase PIKK family		36	34
55	Enzyme	Kinase	Protein Kinase	Atypical protein kinase group	Atypical protein kinase PIKK family	Atypical protein kinase FRAP subfamily	47	43
56	Enzyme	Kinase	Protein Kinase	Atypical protein kinase group	Atypical protein kinase RIO family	Atypical protein kinase RIO1 subfamily	9	8
57	Enzyme	Kinase	Protein Kinase	Atypical protein kinase group	Atypical protein kinase RIO family	Atypical protein kinase RIO2 subfamily	7	7
58	Enzyme	Kinase	Protein Kinase	Atypical protein kinase group	Atypical protein kinase RIO family	Atypical protein kinase RIO3 subfamily	10	9
59	Enzyme	Kinase	Protein Kinase	CAMK protein kinase group			8	8
60	Enzyme	Kinase	Protein Kinase	CAMK protein kinase group	CAMK protein kinase CAMK1 family		26	25
61	Enzyme	Kinase	Protein Kinase	CAMK protein kinase group	CAMK protein kinase CAMK1 family	CAMK protein kinase AMPK subfamily	37	33
62	Enzyme	Kinase	Protein Kinase	CAMK protein kinase group	CAMK protein kinase CAMK1 family	CAMK protein kinase BRSK subfamily	11	11
63	Enzyme	Kinase	Protein Kinase	CAMK protein kinase group	CAMK protein kinase CAMK1 family	CAMK protein kinase CHK1 subfamily	22	21
64	Enzyme	Kinase	Protein Kinase	CAMK protein kinase group	CAMK protein kinase CAMK1 family	CAMK protein kinase LKB subfamily	10	9
65	Enzyme	Kinase	Protein Kinase	CAMK protein kinase group	CAMK protein kinase CAMK1 family	CAMK protein kinase MARK subfamily	64	64
66	Enzyme	Kinase	Protein Kinase	CAMK protein kinase group	CAMK protein kinase CAMK1 family	CAMK protein kinase MELK subfamily	26	24
67	Enzyme	Kinase	Protein Kinase	CAMK protein kinase group	CAMK protein kinase CAMK1 family	CAMK protein kinase NIM1 subfamily	4	4
68	Enzyme	Kinase	Protein Kinase	CAMK protein kinase group	CAMK protein kinase CAMK1 family	CAMK protein kinase NuaK subfamily	45	42
69	Enzyme	Kinase	Protein Kinase	CAMK protein kinase group	CAMK protein kinase CAMK1 family	CAMK protein kinase PASK subfamily	1	1
70	Enzyme	Kinase	Protein Kinase	CAMK protein kinase group	CAMK protein kinase CAMK1 family	CAMK protein kinase QIK subfamily	57	52
71	Enzyme	Kinase	Protein Kinase	CAMK protein kinase group	CAMK protein kinase CAMK2 family		54	55
72	Enzyme	Kinase	Protein Kinase	CAMK protein kinase group	CAMK protein kinase DAPK family		62	61
73	Enzyme	Kinase	Protein Kinase	CAMK protein kinase group	CAMK protein kinase DCAMK1 family		27	24
74	Enzyme	Kinase	Protein Kinase	CAMK protein kinase group	CAMK protein kinase MAPKAPK family	CAMK protein kinase MAPKAPK subfamily	21	21
75	Enzyme	Kinase	Protein Kinase	CAMK protein kinase group	CAMK protein kinase MAPKAPK family	CAMK protein kinase MNK subfamily	36	37
76	Enzyme	Kinase	Protein Kinase	CAMK protein kinase group	CAMK protein kinase MLCK family		53	50
77	Enzyme	Kinase	Protein Kinase	CAMK protein kinase group	CAMK protein kinase PHk family		26	24
78	Enzyme	Kinase	Protein Kinase	CAMK protein kinase group	CAMK protein kinase PIM family		48	51
79	Enzyme	Kinase	Protein Kinase	CAMK protein kinase group	CAMK protein kinase PKD family		72	69
80	Enzyme	Kinase	Protein Kinase	CAMK protein kinase group	CAMK protein kinase RAD53 family		16	16
81	Enzyme	Kinase	Protein Kinase	CAMK protein kinase group	CAMK protein kinase TSSK family		10	10
82	Enzyme	Kinase	Protein Kinase	CAMK protein kinase group	CAMK protein kinase unique family		13	13
83	Enzyme	Kinase	Protein Kinase	CK1 protein kinase group	CK1 protein kinase CK1 family		98	92
84	Enzyme	Kinase	Protein Kinase	CK1 protein kinase group	CK1 protein kinase CK1 family	CK1 protein kinase CK1-a	2	2
85	Enzyme	Kinase	Protein Kinase	CK1 protein kinase group	CK1 protein kinase CK1 family	CK1 protein kinase CK1-g	7	7
86	Enzyme	Kinase	Protein Kinase	CK1 protein kinase group	CK1 protein kinase VRK family		2	2
87	Enzyme	Kinase	Protein Kinase	CMGC protein kinase group			35	32
88	Enzyme	Kinase	Protein Kinase	CMGC protein kinase group	CMGC protein kinase CDK family		6	6
89	Enzyme	Kinase	Protein Kinase	CMGC protein kinase group	CMGC protein kinase CDK family	CMGC protein kinase CDC2 subfamily	85	81
90	Enzyme	Kinase	Protein Kinase	CMGC protein kinase group	CMGC protein kinase CDK family	CMGC protein kinase CDK5 subfamily	27	26
91	Enzyme	Kinase	Protein Kinase	CMGC protein kinase group	CMGC protein kinase CDK family	CMGC protein kinase CDK7 subfamily	40	38
92	Enzyme	Kinase	Protein Kinase	CMGC protein kinase group	CMGC protein kinase CDK family	CMGC protein kinase CDK8 subfamily	18	18
93	Enzyme	Kinase	Protein Kinase	CMGC protein kinase group	CMGC protein kinase CDK family	CMGC protein kinase CDK9 subfamily	23	21
94	Enzyme	Kinase	Protein Kinase	CMGC protein kinase group	CMGC protein kinase CDK family	CMGC protein kinase PCTAIRE	26	24
95	Enzyme	Kinase	Protein Kinase	CMGC protein kinase group	CMGC protein kinase CDK family	CMGC protein kinase PFTAIRE	13	12
96	Enzyme	Kinase	Protein Kinase	CMGC protein kinase group	CMGC protein kinase CDK family	CMGC protein kinase PITSLRE subfamily	13	13
97	Enzyme	Kinase	Protein Kinase	CMGC protein kinase group	CMGC protein kinase CDK family	CMGC protein kinase TAIRE subfamily	24	23
98	Enzyme	Kinase	Protein Kinase	CMGC protein kinase group	CMGC protein kinase CDKL family		13	13

Index	Level1	Level2	Level3	Level4	Level5	Level6	Python_ Workflow	Knime_ Workflow
99	Enzyme	Kinase	Protein Kinase	CMGC protein kinase group	CMGC protein kinase CK subfamily		1	1
100	Enzyme	Kinase	Protein Kinase	CMGC protein kinase group	CMGC protein kinase CLK family		154	146
101	Enzyme	Kinase	Protein Kinase	CMGC protein kinase group	CMGC protein kinase DYRK family		2	2
102	Enzyme	Kinase	Protein Kinase	CMGC protein kinase group	CMGC protein kinase DYRK family	CMGC protein kinase Dyrk1 subfamily	68	62
103	Enzyme	Kinase	Protein Kinase	CMGC protein kinase group	CMGC protein kinase DYRK family	CMGC protein kinase Dyrk2 subfamily	20	20
104	Enzyme	Kinase	Protein Kinase	CMGC protein kinase group	CMGC protein kinase DYRK family	CMGC protein kinase HIPK subfamily	74	72
105	Enzyme	Kinase	Protein Kinase	CMGC protein kinase group	CMGC protein kinase GSK family		95	88
106	Enzyme	Kinase	Protein Kinase	CMGC protein kinase group	CMGC protein kinase MAPK family		16	15
107	Enzyme	Kinase	Protein Kinase	CMGC protein kinase group	CMGC protein kinase MAPK family	CMGC protein kinase ERK subfamily	26	25
108	Enzyme	Kinase	Protein Kinase	CMGC protein kinase group	CMGC protein kinase MAPK family	CMGC protein kinase ERK3	1	1
109	Enzyme	Kinase	Protein Kinase	CMGC protein kinase group	CMGC protein kinase MAPK family	CMGC protein kinase ERK5	15	13
110	Enzyme	Kinase	Protein Kinase	CMGC protein kinase group	CMGC protein kinase MAPK family	CMGC protein kinase JNK subfamily	90	82
111	Enzyme	Kinase	Protein Kinase	CMGC protein kinase group	CMGC protein kinase MAPK family	CMGC protein kinase nmo subfamily	10	10
112	Enzyme	Kinase	Protein Kinase	CMGC protein kinase group	CMGC protein kinase MAPK family	CMGC protein kinase p38 subfamily	83	79
113	Enzyme	Kinase	Protein Kinase	CMGC protein kinase group	CMGC protein kinase RCK family	CMGC protein kinase MAK	19	19
114	Enzyme	Kinase	Protein Kinase	CMGC protein kinase group	CMGC protein kinase SRPK family		29	26
115	Enzyme	Kinase	Protein Kinase	Other protein kinase group			34	32
116	Enzyme	Kinase	Protein Kinase	Other protein kinase group	Other protein kinase AUR family		154	143
117	Enzyme	Kinase	Protein Kinase	Other protein kinase group	Other protein kinase CAMKK family	Other protein kinase Meta subfamily	29	25
118	Enzyme	Kinase	Protein Kinase	Other protein kinase group	Other protein kinase CDC7 family		9	10
119	Enzyme	Kinase	Protein Kinase	Other protein kinase group	Other protein kinase CK2 family		44	43
120	Enzyme	Kinase	Protein Kinase	Other protein kinase group	Other protein kinase Haspin family		1	1
121	Enzyme	Kinase	Protein Kinase	Other protein kinase group	Other protein kinase IKK family		24	22
122	Enzyme	Kinase	Protein Kinase	Other protein kinase group	Other protein kinase NAK family		131	126
123	Enzyme	Kinase	Protein Kinase	Other protein kinase group	Other protein kinase NEK family		39	40
124	Enzyme	Kinase	Protein Kinase	Other protein kinase group	Other protein kinase NEK family	Other protein kinase Nek1	7	7
125	Enzyme	Kinase	Protein Kinase	Other protein kinase group	Other protein kinase NEK family	Other protein kinase Nek11	4	4
126	Enzyme	Kinase	Protein Kinase	Other protein kinase group	Other protein kinase NKF1 family		12	11
127	Enzyme	Kinase	Protein Kinase	Other protein kinase group	Other protein kinase NKF4 family		9	8
128	Enzyme	Kinase	Protein Kinase	Other protein kinase group	Other protein kinase PEK family	Other protein kinase GCN2 subfamily	11	11
129	Enzyme	Kinase	Protein Kinase	Other protein kinase group	Other protein kinase PEK family	Other protein kinase HRI	9	9
130	Enzyme	Kinase	Protein Kinase	Other protein kinase group	Other protein kinase PEK family	Other protein kinase PEK subfamily	3	3
131	Enzyme	Kinase	Protein Kinase	Other protein kinase group	Other protein kinase PEK family	Other protein kinase PKR	9	8
132	Enzyme	Kinase	Protein Kinase	Other protein kinase group	Other protein kinase PLK family		78	74
133	Enzyme	Kinase	Protein Kinase	Other protein kinase group	Other protein kinase PLK family	Other protein kinase PLK2	7	6
134	Enzyme	Kinase	Protein Kinase	Other protein kinase group	Other protein kinase TLK family		7	7
135	Enzyme	Kinase	Protein Kinase	Other protein kinase group	Other protein kinase TOPK family		2	3
136	Enzyme	Kinase	Protein Kinase	Other protein kinase group	Other protein kinase TTK family		21	20
137	Enzyme	Kinase	Protein Kinase	Other protein kinase group	Other protein kinase ULK family		55	52
138	Enzyme	Kinase	Protein Kinase	Other protein kinase group	Other protein kinase WEE family		17	15
139	Enzyme	Kinase	Protein Kinase	Other protein kinase group	Other protein kinase WEE family	Other protein kinase WEE1	5	5
140	Enzyme	Kinase	Protein Kinase	STE protein kinase group			11	10
141	Enzyme	Kinase	Protein Kinase	STE protein kinase group	STE protein kinase STE11 family		51	49
142	Enzyme	Kinase	Protein Kinase	STE protein kinase group	STE protein kinase STE11 family	STE protein kinase ASK	1	1
143	Enzyme	Kinase	Protein Kinase	STE protein kinase group	STE protein kinase STE11 family	STE protein kinase MEKK2	33	29
144	Enzyme	Kinase	Protein Kinase	STE protein kinase group	STE protein kinase STE20 family	STE protein kinase FRAY subfamily	7	6
145	Enzyme	Kinase	Protein Kinase	STE protein kinase group	STE protein kinase STE20 family	STE protein kinase KHS subfamily	115	104
146	Enzyme	Kinase	Protein Kinase	STE protein kinase group	STE protein kinase STE20 family	STE protein kinase MSN subfamily	86	83

Index	Level1	Level2	Level3	Level4	Level5	Level6	Python_ Workflow	Knime_ Workflow
147	Enzyme	Kinase	Protein Kinase	STE protein kinase group	STE protein kinase STE20 family	STE protein kinase MST subfamily	47	44
148	Enzyme	Kinase	Protein Kinase	STE protein kinase group	STE protein kinase STE20 family	STE protein kinase NinaC subfamily	5	5
149	Enzyme	Kinase	Protein Kinase	STE protein kinase group	STE protein kinase STE20 family	STE protein kinase PAKA subfamily	26	25
150	Enzyme	Kinase	Protein Kinase	STE protein kinase group	STE protein kinase STE20 family	STE protein kinase PAKB subfamily	35	34
151	Enzyme	Kinase	Protein Kinase	STE protein kinase group	STE protein kinase STE20 family	STE protein kinase SLK subfamily	88	82
152	Enzyme	Kinase	Protein Kinase	STE protein kinase group	STE protein kinase STE20 family	STE protein kinase TAO subfamily	52	49
153	Enzyme	Kinase	Protein Kinase	STE protein kinase group	STE protein kinase STE20 family	STE protein kinase YSK subfamily	30	28
154	Enzyme	Kinase	Protein Kinase	STE protein kinase group	STE protein kinase STE7 family		119	109
155	Enzyme	Kinase	Protein Kinase	TK protein kinase group	Tyrosine protein kinase Abl family		127	122
156	Enzyme	Kinase	Protein Kinase	TK protein kinase group	Tyrosine protein kinase Ack family		65	54
157	Enzyme	Kinase	Protein Kinase	TK protein kinase group	Tyrosine protein kinase Alk family		26	24
158	Enzyme	Kinase	Protein Kinase	TK protein kinase group	Tyrosine protein kinase Axl family		75	68
159	Enzyme	Kinase	Protein Kinase	TK protein kinase group	Tyrosine protein kinase Csk family		14	14
160	Enzyme	Kinase	Protein Kinase	TK protein kinase group	Tyrosine protein kinase DDR family		80	78
161	Enzyme	Kinase	Protein Kinase	TK protein kinase group	Tyrosine protein kinase EGFR family		178	171
162	Enzyme	Kinase	Protein Kinase	TK protein kinase group	Tyrosine protein kinase Eph family		212	199
163	Enzyme	Kinase	Protein Kinase	TK protein kinase group	Tyrosine protein kinase FGFR family		127	119
164	Enzyme	Kinase	Protein Kinase	TK protein kinase group	Tyrosine protein kinase Fak family		68	61
165	Enzyme	Kinase	Protein Kinase	TK protein kinase group	Tyrosine protein kinase Fer family		52	50
166	Enzyme	Kinase	Protein Kinase	TK protein kinase group	Tyrosine protein kinase InsR family		65	57
167	Enzyme	Kinase	Protein Kinase	TK protein kinase group	Tyrosine protein kinase JakA family		174	164
168	Enzyme	Kinase	Protein Kinase	TK protein kinase group	Tyrosine protein kinase JakB family		1	1
169	Enzyme	Kinase	Protein Kinase	TK protein kinase group	Tyrosine protein kinase Met family		89	86
170	Enzyme	Kinase	Protein Kinase	TK protein kinase group	Tyrosine protein kinase Musk family		13	12
171	Enzyme	Kinase	Protein Kinase	TK protein kinase group	Tyrosine protein kinase PDGFR family		341	330
172	Enzyme	Kinase	Protein Kinase	TK protein kinase group	Tyrosine protein kinase Ret family		87	82
173	Enzyme	Kinase	Protein Kinase	TK protein kinase group	Tyrosine protein kinase Sev family		24	23
174	Enzyme	Kinase	Protein Kinase	TK protein kinase group	Tyrosine protein kinase Src family		453	432
175	Enzyme	Kinase	Protein Kinase	TK protein kinase group	Tyrosine protein kinase Src family	Tyrosine protein kinase Srm	8	8
176	Enzyme	Kinase	Protein Kinase	TK protein kinase group	Tyrosine protein kinase Syk family		25	25
177	Enzyme	Kinase	Protein Kinase	TK protein kinase group	Tyrosine protein kinase Tec family		90	79
178	Enzyme	Kinase	Protein Kinase	TK protein kinase group	Tyrosine protein kinase Tie family		41	41
179	Enzyme	Kinase	Protein Kinase	TK protein kinase group	Tyrosine protein kinase Trk family		107	101
180	Enzyme	Kinase	Protein Kinase	TK protein kinase group	Tyrosine protein kinase VEGFR family		202	195
181	Enzyme	Kinase	Protein Kinase	TKL protein kinase group			17	16
182	Enzyme	Kinase	Protein Kinase	TKL protein kinase group	TKL protein kinase IRAK family		64	62
183	Enzyme	Kinase	Protein Kinase	TKL protein kinase group	TKL protein kinase LISK family	TKL protein kinase LIMK subfamily	37	39
184	Enzyme	Kinase	Protein Kinase	TKL protein kinase group	TKL protein kinase LISK family	TKL protein kinase TESK subfamily	5	5
185	Enzyme	Kinase	Protein Kinase	TKL protein kinase group	TKL protein kinase LRRK family		39	38
186	Enzyme	Kinase	Protein Kinase	TKL protein kinase group	TKL protein kinase MLK family	TKL protein kinase HH498 subfamily	10	10
187	Enzyme	Kinase	Protein Kinase	TKL protein kinase group	TKL protein kinase MLK family	TKL protein kinase ILK subfamily	2	2
188	Enzyme	Kinase	Protein Kinase	TKL protein kinase group	TKL protein kinase MLK family	TKL protein kinase LZK subfamily	11	10
189	Enzyme	Kinase	Protein Kinase	TKL protein kinase group	TKL protein kinase MLK family	TKL protein kinase MLK subfamily	62	62
190	Enzyme	Kinase	Protein Kinase	TKL protein kinase group	TKL protein kinase MLK family	TKL protein kinase TAK1 subfamily	15	14
191	Enzyme	Kinase	Protein Kinase	TKL protein kinase group	TKL protein kinase RAF family		58	59
192	Enzyme	Kinase	Protein Kinase	TKL protein kinase group	TKL protein kinase RIPK family		74	68
193	Enzyme	Kinase	Protein Kinase	TKL protein kinase group	TKL protein kinase STKR family	TKL protein kinase STKR Type 1 subfamily	117	110
194	Enzyme	Kinase	Protein Kinase	TKL protein kinase group	TKL protein kinase STKR family	TKL protein kinase STKR Type 2 subfamily	37	35

Index	Level1	Level2	Level3	Level4	Level5	Level6	Python_ Workflow	Knime_ Workflow
195	Enzyme	Kinase	Protein Kinase	TKL protein kinase group	TKL protein kinase STKR family	TKL protein kinase STKR1	32	30
196	Enzyme	Kinase	Protein kinase regulatory subunit				19	17
197	Enzyme	Ligase					30	24
198	Enzyme	Lyase					458	481
199	Enzyme	Oxidoreductase					610	528
200	Enzyme	Phosphatase					8	9
201	Enzyme	Phosphatase	Protein Phosphatase	Serine/threonine protein phosphatase			2	2
202	Enzyme	Phosphatase	Protein Phosphatase	Serine/threonine/tyrosine protein phosphatase			4	1
203	Enzyme	Phosphatase	Protein Phosphatase	Tyrosine protein phosphatase			9	9
204	Enzyme	Phosphodiesterase					5	5
205	Enzyme	Phosphodiesterase	Phosphodiesterase 1	Phosphodiesterase 1A			1	1
206	Enzyme	Phosphodiesterase	Phosphodiesterase 10	Phosphodiesterase 10A			6	6
207	Enzyme	Phosphodiesterase	Phosphodiesterase 11	Phosphodiesterase 11A			2	2
208	Enzyme	Phosphodiesterase	Phosphodiesterase 2	Phosphodiesterase 2A			1	1
209	Enzyme	Phosphodiesterase	Phosphodiesterase 3	Phosphodiesterase 3A			9	9
210	Enzyme	Phosphodiesterase	Phosphodiesterase 3	Phosphodiesterase 3B			4	4
211	Enzyme	Phosphodiesterase	Phosphodiesterase 4	Phosphodiesterase 4A			12	13
212	Enzyme	Phosphodiesterase	Phosphodiesterase 4	Phosphodiesterase 4B			7	8
213	Enzyme	Phosphodiesterase	Phosphodiesterase 4	Phosphodiesterase 4C			5	6
214	Enzyme	Phosphodiesterase	Phosphodiesterase 4	Phosphodiesterase 4D			19	18
215	Enzyme	Phosphodiesterase	Phosphodiesterase 5	Phosphodiesterase 5A			10	11
216	Enzyme	Phosphodiesterase	Phosphodiesterase 6	Phosphodiesterase 6A			2	2
217	Enzyme	Phosphodiesterase	Phosphodiesterase 6	Phosphodiesterase 6C			2	2
218	Enzyme	Phosphodiesterase	Phosphodiesterase 6	Phosphodiesterase 6D			1	1
219	Enzyme	Phosphodiesterase	Phosphodiesterase 7	Phosphodiesterase 7A			2	2
220	Enzyme	Phosphodiesterase	Phosphodiesterase 9	Phosphodiesterase 9A			2	2
221	Enzyme	Protease	Aspartic protease				1	1
222	Enzyme	Protease	Aspartic protease	Aspartic protease AA clan	Aspartic protease A1A subfamily		27	30
223	Enzyme	Protease	Aspartic protease	Aspartic protease AA clan	Aspartic protease A2A subfamily		14	15
224	Enzyme	Protease	Cysteine protease				1	1
225	Enzyme	Protease	Cysteine protease	Cysteine protease CA clan	Cysteine protease C12 family		2	2
226	Enzyme	Protease	Cysteine protease	Cysteine protease CA clan	Cysteine protease C19 family		2	2
227	Enzyme	Protease	Cysteine protease	Cysteine protease CA clan	Cysteine protease C1A family		34	33
228	Enzyme	Protease	Cysteine protease	Cysteine protease CA clan	Cysteine protease C2 family		3	3
229	Enzyme	Protease	Cysteine protease	Cysteine protease CD clan	Cysteine protease C14 family		3	3
230	Enzyme	Protease	Cysteine protease	Cysteine protease PAC clan	Cysteine protease C3A subfamily		11	13
231	Enzyme	Protease	Metallo protease	Metallo protease MAE clan	Metallo protease M1 family		13	13
232	Enzyme	Protease	Metallo protease	Metallo protease MAE clan	Metallo protease M13 family		5	5
233	Enzyme	Protease	Metallo protease	Metallo protease MAE clan	Metallo protease M2 family		31	30
234	Enzyme	Protease	Metallo protease	Metallo protease MAE clan	Metallo protease M34 family		2	2
235	Enzyme	Protease	Metallo protease	Metallo protease MAM clan	Metallo protease M10A subfamily		67	66
236	Enzyme	Protease	Metallo protease	Metallo protease MAM clan	Metallo protease M12A subfamily		5	5
237	Enzyme	Protease	Metallo protease	Metallo protease MAM clan	Metallo protease M12B subfamily		14	14
238	Enzyme	Protease	Metallo protease	Metallo protease MF clan	Metallo protease M17 family		2	2
239	Enzyme	Protease	Metallo protease	Metallo protease MG clan	Metallo protease M24A subfamily		2	2
240	Enzyme	Protease	Metallo protease	Metallo protease MH clan	Metallo protease M28 family	Metallo protease M28A subfamily	2	1
241	Enzyme	Protease	Metallo protease	Metallo protease MH clan	Metallo protease M28 family	Metallo protease M28B subfamily	3	3
242	Enzyme	Protease	Metallo protease	Metallo protease MJ clan	Metallo protease M19 family		1	
243	Enzyme	Protease	Serine protease				17	16
244	Enzyme	Protease	Serine protease	Serine protease PA clan	Serine protease S1A subfamily		40	37
245	Enzyme	Protease	Serine protease	Serine protease SB clan	Serine protease S8A subfamily		2	2
246	Enzyme	Protease	Serine protease	Serine protease SC clan	Serine protease S28 family		4	4
247	Enzyme	Protease	Serine protease	Serine protease SC clan	Serine protease S33 family		8	8
248	Enzyme	Protease	Serine protease	Serine protease SC clan	Serine protease S9A subfamily		1	1
249	Enzyme	Protease	Serine protease	Serine protease SC clan	Serine protease S9B subfamily		35	34
250	Enzyme	Protease	Threonine protease	Threonine protease PBT clan	Threonine protease T1A subfamily		25	23
251	Enzyme	Protease	Threonine protease	Threonine protease PBT clan	Threonine protease T1B subfamily		2	2
252	Enzyme	Transferase					517	467
253	Epigenetic regulator	Eraser	Histone deacetylase	HDAC class I			113	106
254	Epigenetic regulator	Eraser	Histone deacetylase	HDAC class III			10	9

Index	Level1	Level2	Level3	Level4	Level5	Level6	Python_ Workflow	Knime_ Workflow
255	Epigenetic regulator	Eraser	Histone deacetylase	HDAC class IIa			61	55
256	Epigenetic regulator	Eraser	Histone deacetylase	HDAC class IIb			101	94
257	Epigenetic regulator	Eraser	Histone deacetylase	HDAC class IV			16	15
258	Epigenetic regulator	Eraser	Lysine demethylase	Jumonji domain-containing			26	20
259	Epigenetic regulator	Eraser	Lysine demethylase	Lysine-specific demethylase			16	13
260	Epigenetic regulator	Reader					1	1
261	Epigenetic regulator	Reader	Bromodomain				81	71
262	Epigenetic regulator	Reader	Methyl-lysine/arginine binding protein	MBT domain			1	1
263	Epigenetic regulator	Reader	Methyl-lysine/arginine binding protein	PWWP domain			2	2
264	Epigenetic regulator	Reader	Methyl-lysine/arginine binding protein	Tudor domain			1	1
265	Epigenetic regulator	Writer	DNA methyltransferase				6	5
266	Epigenetic regulator	Writer	Histone acetyltransferase	SRC family			2	1
267	Epigenetic regulator	Writer	Histone acetyltransferase	p300/CBP family			7	6
268	Epigenetic regulator	Writer	Protein methyltransferase				36	25
269	Epigenetic regulator	Writer	Protein methyltransferase	Protein arginine methyltransferase			4	4
270	Epigenetic regulator	Writer	Protein methyltransferase	SET domain			8	4
271	Ion channel	Ligand-gated ion channel	5HT3 receptor				33	32
272	Ion channel	Ligand-gated ion channel	Epithelial sodium channel				1	1
273	Ion channel	Ligand-gated ion channel	GABA-A receptor				58	54
274	Ion channel	Ligand-gated ion channel	Glycine receptor				4	3
275	Ion channel	Ligand-gated ion channel	Ionotropic glutamate receptor	AMPA receptor			17	17
276	Ion channel	Ligand-gated ion channel	Ionotropic glutamate receptor	Kainate receptor			20	20
277	Ion channel	Ligand-gated ion channel	Ionotropic glutamate receptor	NMDA receptor			25	23
278	Ion channel	Ligand-gated ion channel	Nicotinic acetylcholine receptor				2	
279	Ion channel	Ligand-gated ion channel	Nicotinic acetylcholine receptor	Nicotinic acetylcholine receptor alpha subunit			22	20
280	Ion channel	Ligand-gated ion channel	Nicotinic acetylcholine receptor	Nicotinic acetylcholine receptor beta subunit			2	1
281	Ion channel	Ligand-gated ion channel	Nicotinic acetylcholine receptor	Nicotinic acetylcholine receptor epsilon subunit			1	1
282	Ion channel	Ligand-gated ion channel	P2X receptor				34	26
283	Ion channel	Other ion channel	Chloride channel	Calcium-activated chloride channel			2	2
284	Ion channel	Other ion channel	Chloride channel	Cystic fibrosis transmembrane conductance regulator			2	2
285	Ion channel	Other ion channel	Miscellaneous ion channel	Bcl-2 family			11	11
286	Ion channel	Other ion channel	Miscellaneous ion channel	Presenilin ER Ca2+ leak channel family			1	1
287	Ion channel	Other ion channel	Miscellaneous ion channel	Type A influenza virus matrix-2 channel family			4	4
288	Ion channel	Other ion channel	Mitochondrial and plastid porin family	Voltage-dependent anion-selective channel			1	1
289	Ion channel	Other ion channel	Pore-forming toxins (proteins and peptides)				2	2
290	Ion channel	Voltage-gated ion channel	Potassium channels	Calcium-activated potassium channel			11	9
291	Ion channel	Voltage-gated ion channel	Potassium channels	Inwardly rectifying potassium channel			2	2
292	Ion channel	Voltage-gated ion channel	Potassium channels	Two-pore domain potassium channel			7	7
293	Ion channel	Voltage-gated ion channel	Potassium channels	Voltage-gated potassium channel			139	127
294	Ion channel	Voltage-gated ion channel	Transient receptor potential channel				63	62
295	Ion channel	Voltage-gated ion channel	Voltage-gated calcium channel				53	44
296	Ion channel	Voltage-gated ion channel	Voltage-gated sodium channel				34	34
297	Membrane receptor						219	207
298	Membrane receptor	Family A G protein-coupled receptor					11	10
299	Membrane receptor	Family A G protein-coupled receptor	Peptide receptor (family A GPCR)	Anaphylatoxin receptor family	Anaphylatoxin receptor		1	1
300	Membrane receptor	Family A G protein-coupled receptor	Peptide receptor (family A GPCR)	Chemokine receptor	CC chemokine receptor		31	26

Index	Level1	Level2	Level3	Level4	Level5	Level6	Python_ Workflow	Knime_ Workflow
301	Membrane receptor	Family A G protein-coupled receptor	Peptide receptor (family A GPCR)	Chemokine receptor	CX3C chemokine receptor		1	1
302	Membrane receptor	Family A G protein-coupled receptor	Peptide receptor (family A GPCR)	Chemokine receptor	CXC chemokine receptor		21	20
303	Membrane receptor	Family A G protein-coupled receptor	Peptide receptor (family A GPCR)	N-formyl methionyl peptide receptor			1	1
304	Membrane receptor	Family A G protein-coupled receptor	Peptide receptor (family A GPCR)	N-formyl methionyl peptide receptor	N-formyl methionyl peptide receptor		1	1
305	Membrane receptor	Family A G protein-coupled receptor	Peptide receptor (family A GPCR)	Protease-activated receptor	Protease-activated receptor		5	5
306	Membrane receptor	Family A G protein-coupled receptor	Peptide receptor (family A GPCR)	Relaxin-like peptide receptor (family A GPCR)	Relaxin receptor		1	1
307	Membrane receptor	Family A G protein-coupled receptor	Peptide receptor (family A GPCR)	Short peptide receptor (family A GPCR)	Angiotensin receptor		43	39
308	Membrane receptor	Family A G protein-coupled receptor	Peptide receptor (family A GPCR)	Short peptide receptor (family A GPCR)	Bradykinin receptor		1	1
309	Membrane receptor	Family A G protein-coupled receptor	Peptide receptor (family A GPCR)	Short peptide receptor (family A GPCR)	Cholecystokinin receptor		11	5
310	Membrane receptor	Family A G protein-coupled receptor	Peptide receptor (family A GPCR)	Short peptide receptor (family A GPCR)	Endothelin receptor		30	37
311	Membrane receptor	Family A G protein-coupled receptor	Peptide receptor (family A GPCR)	Short peptide receptor (family A GPCR)	GRP-related receptor		14	14
312	Membrane receptor	Family A G protein-coupled receptor	Peptide receptor (family A GPCR)	Short peptide receptor (family A GPCR)	Galanin receptor		2	3
313	Membrane receptor	Family A G protein-coupled receptor	Peptide receptor (family A GPCR)	Short peptide receptor (family A GPCR)	GnRH receptor		4	8
314	Membrane receptor	Family A G protein-coupled receptor	Peptide receptor (family A GPCR)	Short peptide receptor (family A GPCR)	MCH receptor		3	3
315	Membrane receptor	Family A G protein-coupled receptor	Peptide receptor (family A GPCR)	Short peptide receptor (family A GPCR)	Melanocortin receptor		7	7
316	Membrane receptor	Family A G protein-coupled receptor	Peptide receptor (family A GPCR)	Short peptide receptor (family A GPCR)	Motilin receptor		4	4
317	Membrane receptor	Family A G protein-coupled receptor	Peptide receptor (family A GPCR)	Short peptide receptor (family A GPCR)	Neurokinin receptor		38	45
318	Membrane receptor	Family A G protein-coupled receptor	Peptide receptor (family A GPCR)	Short peptide receptor (family A GPCR)	Neuropeptide Y receptor		10	4
319	Membrane receptor	Family A G protein-coupled receptor	Peptide receptor (family A GPCR)	Short peptide receptor (family A GPCR)	Neuropeptide receptor		8	8
320	Membrane receptor	Family A G protein-coupled receptor	Peptide receptor (family A GPCR)	Short peptide receptor (family A GPCR)	Neurotensin receptor		3	3
321	Membrane receptor	Family A G protein-coupled receptor	Peptide receptor (family A GPCR)	Short peptide receptor (family A GPCR)	Opioid receptor		197	167
322	Membrane receptor	Family A G protein-coupled receptor	Peptide receptor (family A GPCR)	Short peptide receptor (family A GPCR)	Orexin receptor		12	15
323	Membrane receptor	Family A G protein-coupled receptor	Peptide receptor (family A GPCR)	Short peptide receptor (family A GPCR)	Somatostatin receptor		8	12
324	Membrane receptor	Family A G protein-coupled receptor	Peptide receptor (family A GPCR)	Short peptide receptor (family A GPCR)	Vasopressin and oxytocin receptor		38	35
325	Membrane receptor	Family A G protein-coupled receptor	Small molecule receptor (family A GPCR)				1	1
326	Membrane receptor	Family A G protein-coupled receptor	Small molecule receptor (family A GPCR)	Carboxylic acid receptor	Hydroxycarboxylic acid receptor		9	9
327	Membrane receptor	Family A G protein-coupled receptor	Small molecule receptor (family A GPCR)	Carboxylic acid receptor	Kynurenic acid receptor		9	8
328	Membrane receptor	Family A G protein-coupled receptor	Small molecule receptor (family A GPCR)	Carboxylic acid receptor	Oxoglutarate receptor		1	1
329	Membrane receptor	Family A G protein-coupled receptor	Small molecule receptor (family A GPCR)	Lipid-like ligand receptor (family A GPCR)			8	8
330	Membrane receptor	Family A G protein-coupled receptor	Small molecule receptor (family A GPCR)	Lipid-like ligand receptor (family A GPCR)	Cannabinoid receptor		54	49
331	Membrane receptor	Family A G protein-coupled receptor	Small molecule receptor (family A GPCR)	Lipid-like ligand receptor (family A GPCR)	EDG receptor		27	26
332	Membrane receptor	Family A G protein-coupled receptor	Small molecule receptor (family A GPCR)	Lipid-like ligand receptor (family A GPCR)	Free fatty acid receptor		16	16
333	Membrane receptor	Family A G protein-coupled receptor	Small molecule receptor (family A GPCR)	Lipid-like ligand receptor (family A GPCR)	Leukotriene receptor		17	17

Index	Level1	Level2	Level3	Level4	Level5	Level6	Python_ Workflow	Knime_ Workflow
334	Membrane receptor	Family A G protein-coupled receptor	Small molecule receptor (family A GPCR)	Lipid-like ligand receptor (family A GPCR)	Lysophosphatidylinositol receptor		3	3
335	Membrane receptor	Family A G protein-coupled receptor	Small molecule receptor (family A GPCR)	Lipid-like ligand receptor (family A GPCR)	PAF receptor		4	4
336	Membrane receptor	Family A G protein-coupled receptor	Small molecule receptor (family A GPCR)	Lipid-like ligand receptor (family A GPCR)	Prostanoid receptor		56	55
337	Membrane receptor	Family A G protein-coupled receptor	Small molecule receptor (family A GPCR)	Lipid-like ligand receptor (family A GPCR)	Steroid-like ligand receptor		8	8
338	Membrane receptor	Family A G protein-coupled receptor	Small molecule receptor (family A GPCR)	Monoamine receptor	Acetylcholine receptor		453	447
339	Membrane receptor	Family A G protein-coupled receptor	Small molecule receptor (family A GPCR)	Monoamine receptor	Adrenergic receptor		855	806
340	Membrane receptor	Family A G protein-coupled receptor	Small molecule receptor (family A GPCR)	Monoamine receptor	Dopamine receptor		558	540
341	Membrane receptor	Family A G protein-coupled receptor	Small molecule receptor (family A GPCR)	Monoamine receptor	Histamine receptor		265	257
342	Membrane receptor	Family A G protein-coupled receptor	Small molecule receptor (family A GPCR)	Monoamine receptor	Serotonin receptor		890	870
343	Membrane receptor	Family A G protein-coupled receptor	Small molecule receptor (family A GPCR)	Monoamine receptor	Trace amine receptor		5	5
344	Membrane receptor	Family A G protein-coupled receptor	Small molecule receptor (family A GPCR)	Monoamine-derivative receptor (family A GPCR)	Melatonin receptor		20	18
345	Membrane receptor	Family A G protein-coupled receptor	Small molecule receptor (family A GPCR)	Nucleotide-like receptor (family A GPCR)	Adenosine receptor		214	219
346	Membrane receptor	Family A G protein-coupled receptor	Small molecule receptor (family A GPCR)	Nucleotide-like receptor (family A GPCR)	Purine receptor		12	9
347	Membrane receptor	Family B G protein-coupled receptor	Peptide receptor (family B GPCR)	Calcitonin-like receptor	Calcitonin gene-related peptide receptor		6	6
348	Membrane receptor	Family B G protein-coupled receptor	Peptide receptor (family B GPCR)	Corticotropin releasing factor receptor	Corticotropin releasing factor receptor		9	8
349	Membrane receptor	Family B G protein-coupled receptor	Peptide receptor (family B GPCR)	Glucagon-like receptor	Glucagon receptor		8	8
350	Membrane receptor	Family B G protein-coupled receptor	Peptide receptor (family B GPCR)	Glucagon-like receptor	Glucagon-like peptide receptor		2	2
351	Membrane receptor	Family B G protein-coupled receptor	Peptide receptor (family B GPCR)	Glucagon-like receptor	Secretin receptor receptor		1	1
352	Membrane receptor	Family C G protein-coupled receptor	Ion receptor (family C GPCR)	Calcium sensing receptor	Calcium sensing receptor		6	6
353	Membrane receptor	Family C G protein-coupled receptor	Small molecule receptor (family C GPCR)	Neurotransmitter receptor (family C GPCR)	GABA-B receptor		1	1
354	Membrane receptor	Family C G protein-coupled receptor	Small molecule receptor (family C GPCR)	Neurotransmitter receptor (family C GPCR)	Metabotropic glutamate receptor		60	57
355	Membrane receptor	Frizzled family G protein-coupled receptor	Smoothened receptor (frizzled family GPCR)				17	17
356	Membrane receptor	Taste family G protein-coupled receptor	Taste receptor (taste family GPCR)				26	26
357	Membrane receptor	Toll-like and Il-1 receptors					10	10
358	Other cytosolic protein						139	128
359	Other membrane protein						39	31
360	Other nuclear protein						15	13
361	Secreted protein						75	63
362	Structural protein						22	21
363	Surface antigen						8	5
364	Transcription factor						32	28
365	Transcription factor	Nuclear receptor	Nuclear hormone receptor subfamily 0	Nuclear hormone receptor subfamily 0 group B	Nuclear hormone receptor subfamily 0 group B member 1		1	1
366	Transcription factor	Nuclear receptor	Nuclear hormone receptor subfamily 1	Nuclear hormone receptor subfamily 1 group A	Nuclear hormone receptor subfamily 1 group A member 1		3	2
367	Transcription factor	Nuclear receptor	Nuclear hormone receptor subfamily 1	Nuclear hormone receptor subfamily 1 group A	Nuclear hormone receptor subfamily 1 group A member 2		4	2
368	Transcription factor	Nuclear receptor	Nuclear hormone receptor subfamily 1	Nuclear hormone receptor subfamily 1 group B	Nuclear hormone receptor subfamily 1 group B member 1		12	12
369	Transcription factor	Nuclear receptor	Nuclear hormone receptor subfamily 1	Nuclear hormone receptor subfamily 1 group B	Nuclear hormone receptor subfamily 1 group B member 2		15	15
370	Transcription factor	Nuclear receptor	Nuclear hormone receptor subfamily 1	Nuclear hormone receptor subfamily 1 group B	Nuclear hormone receptor subfamily 1 group B member 3		12	12
371	Transcription factor	Nuclear receptor	Nuclear hormone receptor subfamily 1	Nuclear hormone receptor subfamily 1 group C	Nuclear hormone receptor subfamily 1 group C member 1		17	14

Index	Level1	Level2	Level3	Level4	Level5	Level6	Python_ Workflow	Knime_ Workflow
372	Transcription factor	Nuclear receptor	Nuclear hormone receptor subfamily 1	Nuclear hormone receptor subfamily 1 group C	Nuclear hormone receptor subfamily 1 group C member 2		12	12
373	Transcription factor	Nuclear receptor	Nuclear hormone receptor subfamily 1	Nuclear hormone receptor subfamily 1 group C	Nuclear hormone receptor subfamily 1 group C member 3		26	18
374	Transcription factor	Nuclear receptor	Nuclear hormone receptor subfamily 1	Nuclear hormone receptor subfamily 1 group F			1	1
375	Transcription factor	Nuclear receptor	Nuclear hormone receptor subfamily 1	Nuclear hormone receptor subfamily 1 group F	Nuclear hormone receptor subfamily 1 group F member 1		2	2
376	Transcription factor	Nuclear receptor	Nuclear hormone receptor subfamily 1	Nuclear hormone receptor subfamily 1 group F	Nuclear hormone receptor subfamily 1 group F member 3		4	4
377	Transcription factor	Nuclear receptor	Nuclear hormone receptor subfamily 1	Nuclear hormone receptor subfamily 1 group H			1	1
378	Transcription factor	Nuclear receptor	Nuclear hormone receptor subfamily 1	Nuclear hormone receptor subfamily 1 group H	Nuclear hormone receptor subfamily 1 group H member 3		8	8
379	Transcription factor	Nuclear receptor	Nuclear hormone receptor subfamily 1	Nuclear hormone receptor subfamily 1 group H	Nuclear hormone receptor subfamily 1 group H member 4		8	9
380	Transcription factor	Nuclear receptor	Nuclear hormone receptor subfamily 1	Nuclear hormone receptor subfamily 1 group I	Nuclear hormone receptor subfamily 1 group I member 1		13	11
381	Transcription factor	Nuclear receptor	Nuclear hormone receptor subfamily 1	Nuclear hormone receptor subfamily 1 group I	Nuclear hormone receptor subfamily 1 group I member 2		22	17
382	Transcription factor	Nuclear receptor	Nuclear hormone receptor subfamily 1	Nuclear hormone receptor subfamily 1 group I	Nuclear hormone receptor subfamily 1 group I member 3		6	6
383	Transcription factor	Nuclear receptor	Nuclear hormone receptor subfamily 2	Nuclear hormone receptor subfamily 2 group B	Nuclear hormone receptor subfamily 2 group B member 1		7	7
384	Transcription factor	Nuclear receptor	Nuclear hormone receptor subfamily 2	Nuclear hormone receptor subfamily 2 group B	Nuclear hormone receptor subfamily 2 group B member 2		5	5
385	Transcription factor	Nuclear receptor	Nuclear hormone receptor subfamily 2	Nuclear hormone receptor subfamily 2 group B	Nuclear hormone receptor subfamily 2 group B member 3		4	4
386	Transcription factor	Nuclear receptor	Nuclear hormone receptor subfamily 2	Nuclear hormone receptor subfamily 2 group E	Nuclear hormone receptor subfamily 2 group E member 1		1	1
387	Transcription factor	Nuclear receptor	Nuclear hormone receptor subfamily 3	Nuclear hormone receptor subfamily 3 group A	Nuclear hormone receptor subfamily 3 group A member 1		40	29
388	Transcription factor	Nuclear receptor	Nuclear hormone receptor subfamily 3	Nuclear hormone receptor subfamily 3 group A	Nuclear hormone receptor subfamily 3 group A member 2		33	26
389	Transcription factor	Nuclear receptor	Nuclear hormone receptor subfamily 3	Nuclear hormone receptor subfamily 3 group B	Nuclear hormone receptor subfamily 3 group B member 1		3	2
390	Transcription factor	Nuclear receptor	Nuclear hormone receptor subfamily 3	Nuclear hormone receptor subfamily 3 group B	Nuclear hormone receptor subfamily 3 group B member 2		2	1
391	Transcription factor	Nuclear receptor	Nuclear hormone receptor subfamily 3	Nuclear hormone receptor subfamily 3 group B	Nuclear hormone receptor subfamily 3 group B member 3		3	3
392	Transcription factor	Nuclear receptor	Nuclear hormone receptor subfamily 3	Nuclear hormone receptor subfamily 3 group C	Nuclear hormone receptor subfamily 3 group C member 1		45	23
393	Transcription factor	Nuclear receptor	Nuclear hormone receptor subfamily 3	Nuclear hormone receptor subfamily 3 group C	Nuclear hormone receptor subfamily 3 group C member 2		9	6
394	Transcription factor	Nuclear receptor	Nuclear hormone receptor subfamily 3	Nuclear hormone receptor subfamily 3 group C	Nuclear hormone receptor subfamily 3 group C member 3		66	44
395	Transcription factor	Nuclear receptor	Nuclear hormone receptor subfamily 3	Nuclear hormone receptor subfamily 3 group C	Nuclear hormone receptor subfamily 3 group C member 4		68	54
396	Transcription factor	Nuclear receptor	Nuclear hormone receptor subfamily 4	Nuclear hormone receptor subfamily 4 group A	Nuclear hormone receptor subfamily 4 group A member 2		2	1
397	Transcription factor	Nuclear receptor	Nuclear hormone receptor subfamily 4	Nuclear hormone receptor subfamily 4 group A	Nuclear hormone receptor subfamily 4 group A member 3		1	
398	Transcription factor	Nuclear receptor	Nuclear hormone receptor subfamily 5	Nuclear hormone receptor subfamily 5 group A	Nuclear hormone receptor subfamily 5 group A member 1		2	2
399	Transcription factor	Nuclear receptor	Nuclear hormone receptor subfamily 5	Nuclear hormone receptor subfamily 5 group A	Nuclear hormone receptor subfamily 5 group A member 2		1	1
400	Transporter						4	2
401	Transporter	Electrochemical transporter	SLC superfamily of solute carriers	SLC01 family of amino acid transporters			2	2
402	Transporter	Electrochemical transporter	SLC superfamily of solute carriers	SLC02 family of hexose and sugar alcohol transporters			4	4
403	Transporter	Electrochemical transporter	SLC superfamily of solute carriers	SLC03 and SLC07 families of heteromeric amino acid transporters (HATs)	SLC07 Cationic amino acid transporter/glycoprotein-associated family		2	1

Index	Level1	Level2	Level3	Level4	Level5	Level6	Python_ Workflow	Knime_ Workflow
404	Transporter	Electrochemical transporter	SLC superfamily of solute carriers	SLC05 family of sodium-dependent glucose transporters			9	13
405	Transporter	Electrochemical transporter	SLC superfamily of solute carriers	SLC06 neurotransmitter transporter family			350	325
406	Transporter	Electrochemical transporter	SLC superfamily of solute carriers	SLC09 family of sodium/hydrogen exchangers			11	11
407	Transporter	Electrochemical transporter	SLC superfamily of solute carriers	SLC10 family of sodium-bile acid co-transporters			3	3
408	Transporter	Electrochemical transporter	SLC superfamily of solute carriers	SLC12 family of cation-coupled chloride transporters			1	1
409	Transporter	Electrochemical transporter	SLC superfamily of solute carriers	SLC16 family of monocarboxylate transporters			8	5
410	Transporter	Electrochemical transporter	SLC superfamily of solute carriers	SLC17 phosphate and organic anion transporter family			1	
411	Transporter	Electrochemical transporter	SLC superfamily of solute carriers	SLC18 family of vesicular amine transporters			28	23
412	Transporter	Electrochemical transporter	SLC superfamily of solute carriers	SLC19 family of vitamin transporters			4	2
413	Transporter	Electrochemical transporter	SLC superfamily of solute carriers	SLC21/SLCO family of organic anion transporting polypeptides			3	2
414	Transporter	Electrochemical transporter	SLC superfamily of solute carriers	SLC22 family of organic cation and anion transporters			23	19
415	Transporter	Electrochemical transporter	SLC superfamily of solute carriers	SLC25 family of mitochondrial transporters			3	3
416	Transporter	Electrochemical transporter	SLC superfamily of solute carriers	SLC28 and SLC29 families of nucleoside transporters	SLC29 Facilitative nucleoside transporter family		3	4
417	Transporter	Electrochemical transporter	SLC superfamily of solute carriers	SLC46 family of folate transporters			4	2
418	Transporter	Electrochemical transporter	SLC superfamily of solute carriers	SLC47 family of multidrug and toxin extrusion transporters			14	14
419	Transporter	Primary active transporter	ATP-binding cassette	ABCB subfamily			33	24
420	Transporter	Primary active transporter	ATP-binding cassette	ABCC subfamily			2	2
421	Transporter	Primary active transporter	ATP-binding cassette	ABCG subfamily			18	16
422	Transporter	Primary active transporter	Endoplasmic reticular retrotranslocon family				3	3
423	Transporter	Primary active transporter	Oxidoreduction-driven transporters				3	3
424	Transporter	Primary active transporter	P-type ATPase	Hydrogen potassium ATPase			2	2
425	Transporter	Primary active transporter	P-type ATPase	Sodium potassium ATPase			1	1
426	Transporter	Transmembrane 1-electron transfer carriers					5	8
427	Unclassified protein						197	181
428	[]						2	2
