# Supplementary figures and images for: From library to landscape: integrative annotation workflows for compound libraries in drug repurposing
Source: Database (Oxford). 2025 Dec 9;2025:baaf081. doi: 10.1093/database/baaf081 (PMC12687465; doi:10.1093/database/baaf081)

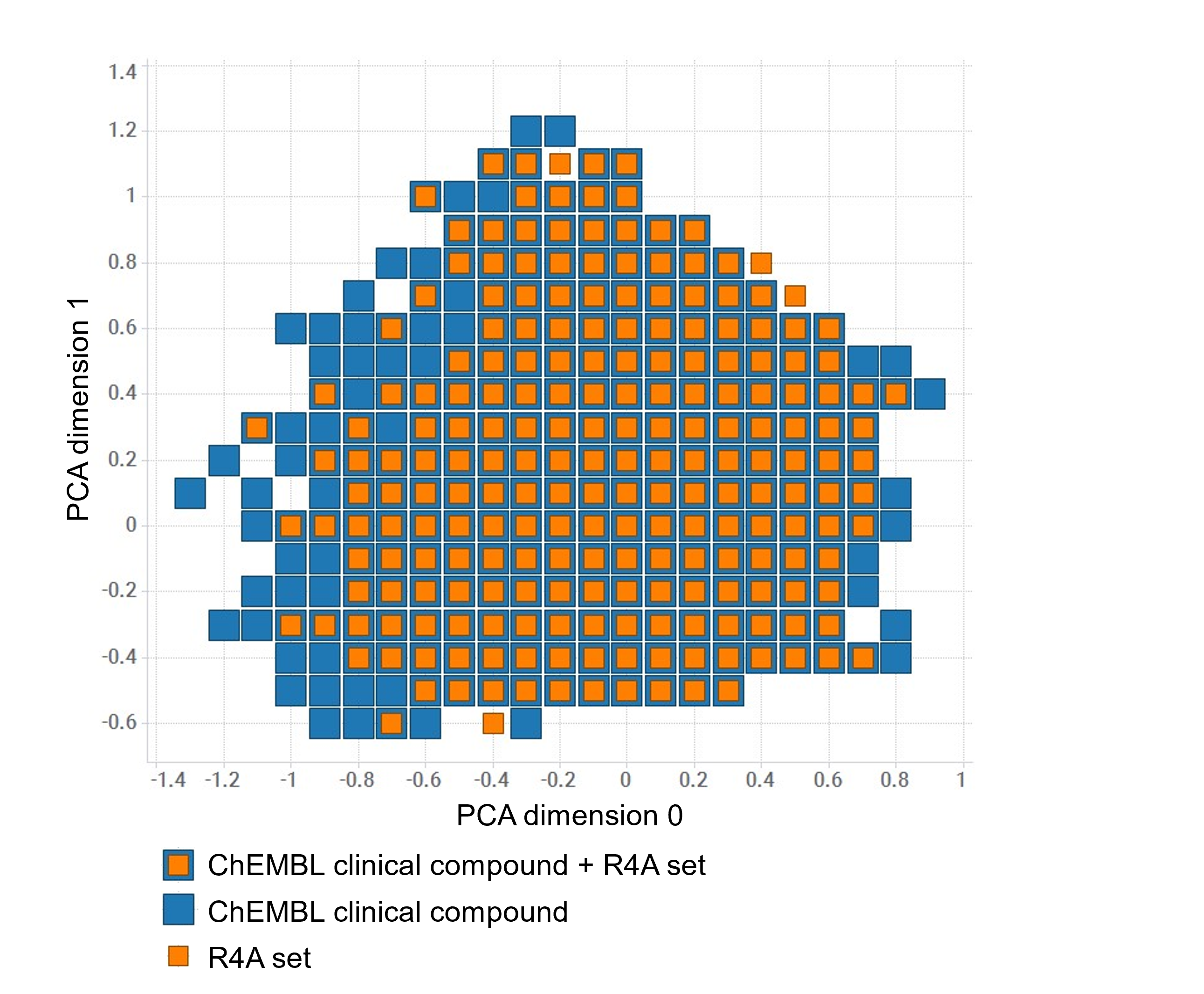

Supplement: baaf081_Supplemental_Files [file baaf081_supplemental_files.zip › FigureS2.tif]

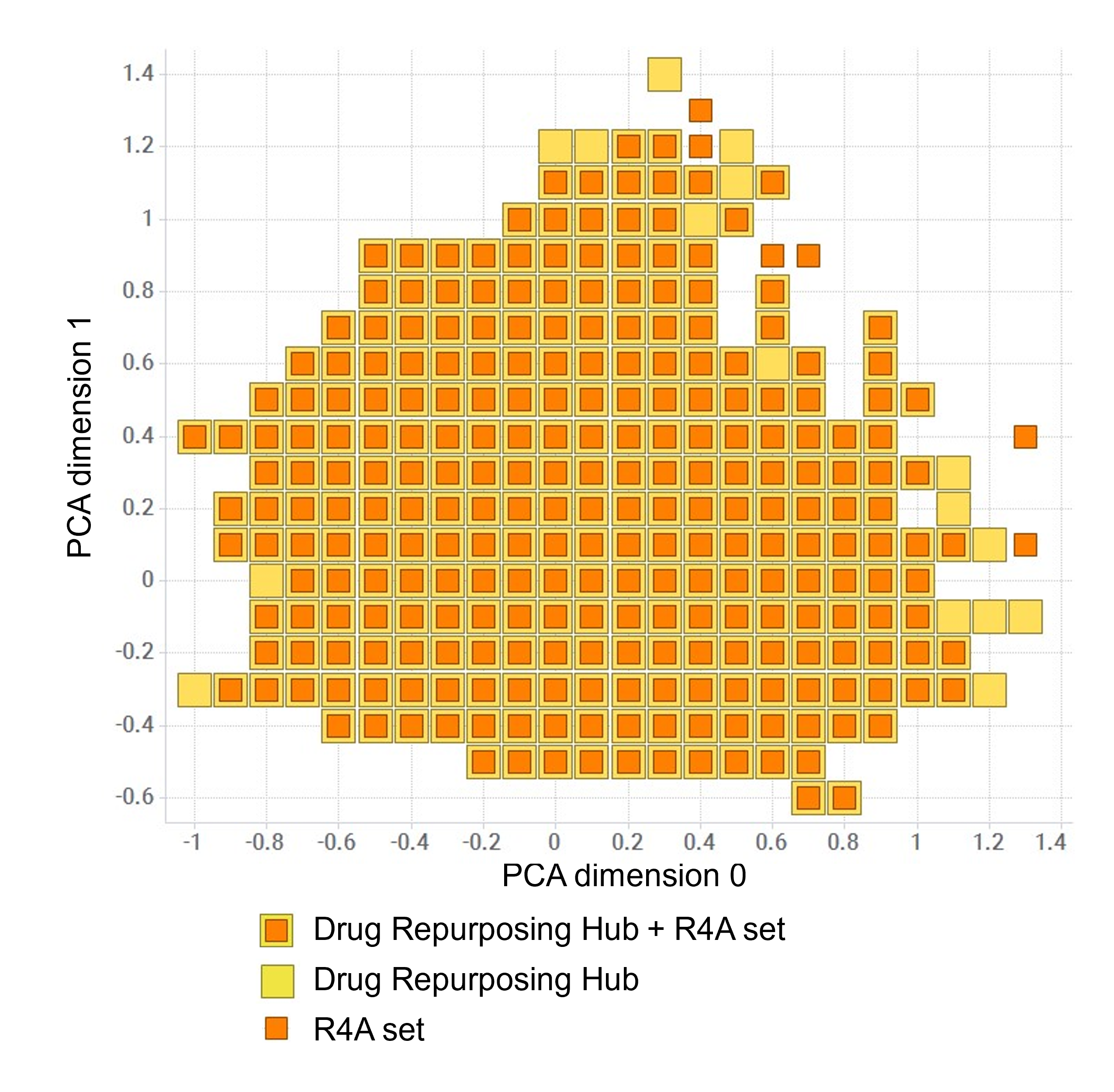

Supplement: baaf081_Supplemental_Files [file baaf081_supplemental_files.zip › FigureS3.tif]

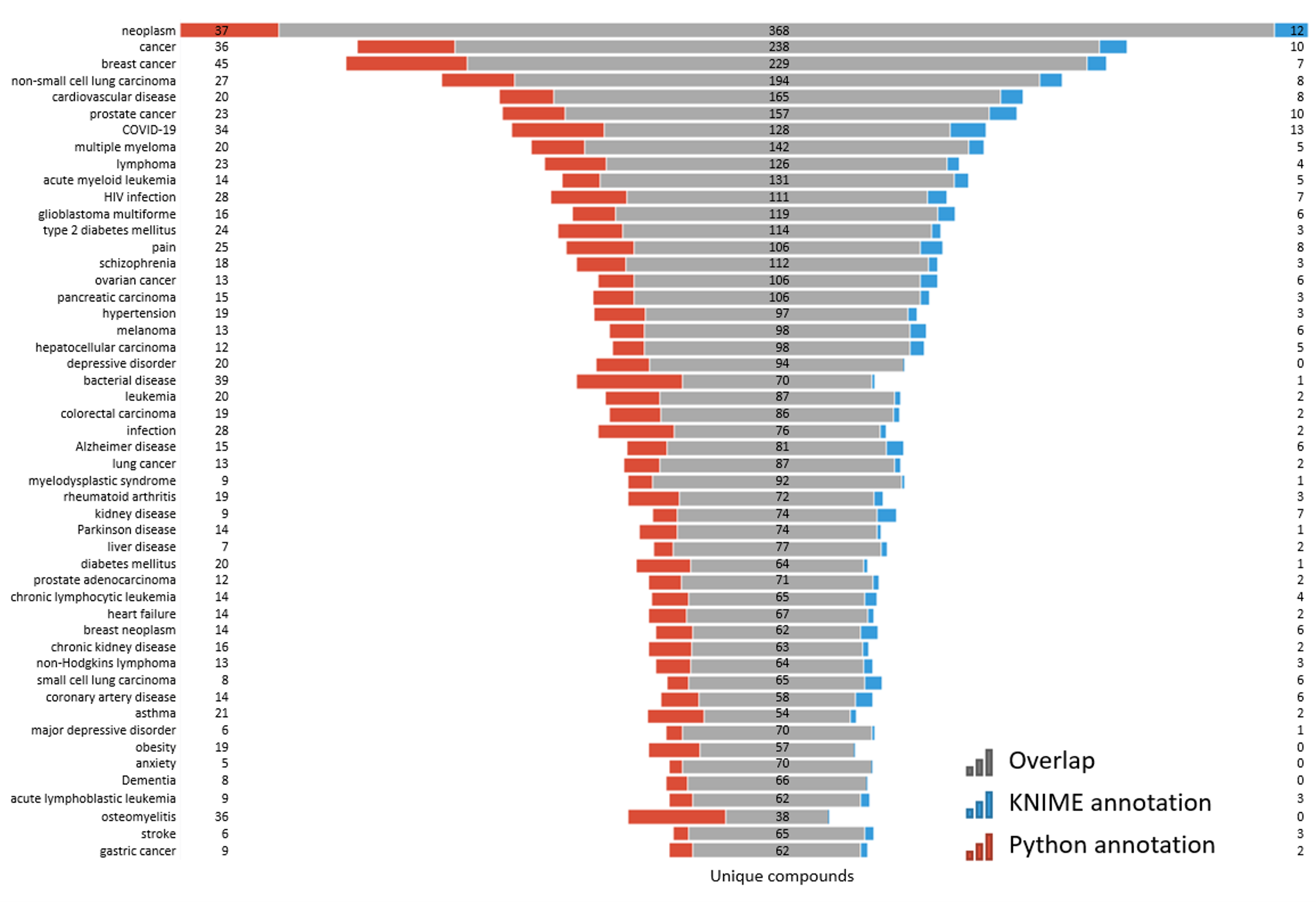

Supplement: baaf081_Supplemental_Files [file baaf081_supplemental_files.zip › FigureS4.tif]

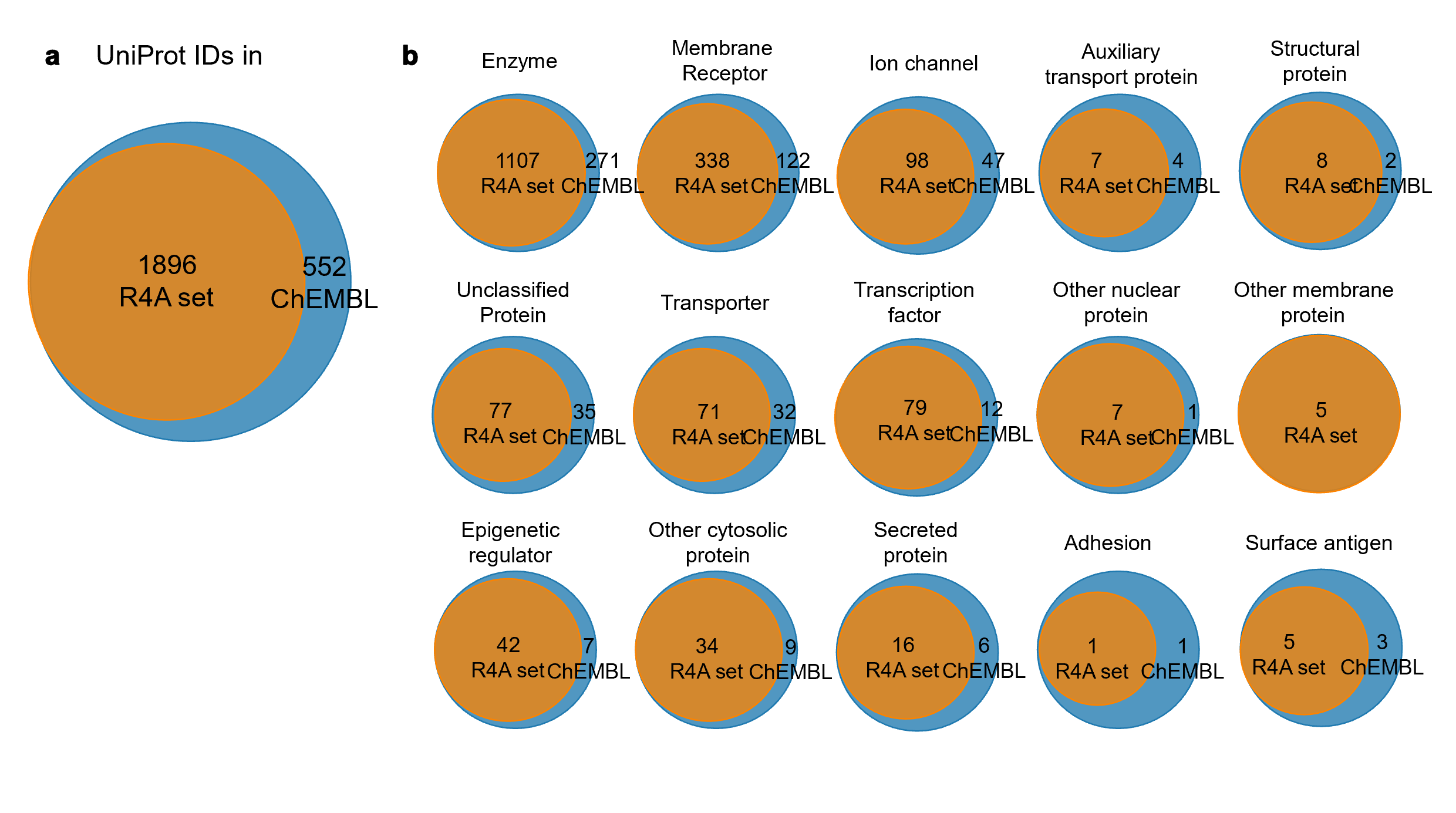

Supplement: baaf081_Supplemental_Files [file baaf081_supplemental_files.zip › FigureS5.tif]
